# Quantitative trait loci analysis reveals candidate genes implicated in regulating functional deficit and CNS vascular permeability in CD8 T cell-initiated blood–brain barrier disruption

**DOI:** 10.1186/1471-2164-14-678

**Published:** 2013-10-03

**Authors:** Holly L Johnson, Lisa M Hanson, Yi Chen, Allan J Bieber, Russell J Buono, Thomas N Ferraro, Istvan Pirko, Aaron J Johnson

**Affiliations:** 1 Department of Neurology, Mayo Clinic, Rochester, MN, USA; 2 Department of Immunology, Mayo Clinic, Rochester, MN, USA; 3 Neurobiology of Disease Graduate Program, Mayo Graduate School, Rochester, MN, USA; 4 Department of Neurology, University of Cincinnati, Cincinnati, OH, USA; 5 Department of Neuroscience, Cooper Medical School of Rowan University, Camden, NJ, USA; 6 Department of Psychiatry, University of Pennsylvania, Philadelphia, PA, USA

**Keywords:** Quantitative trait loci (QTL), Single nucleotide polymorphism (SNP), Blood–brain barrier (BBB), Theiler’s murine encephalomyelitis virus (TMEV), Peptide-induced fatal syndrome (PIFS), CD8 T cell, CNS vascular permeability

## Abstract

**Background:**

Blood–brain barrier (BBB) disruption is an integral feature of numerous neurological disorders. However, there is a relative lack of knowledge regarding the underlying molecular mechanisms of immune-mediated BBB disruption. We have previously shown that CD8 T cells and perforin play critical roles in initiating altered permeability of the BBB in the peptide-induced fatal syndrome (PIFS) model developed by our laboratory. Additionally, despite having indistinguishable CD8 T cell responses, C57BL/6J (B6) mice are highly susceptible to PIFS, exhibiting functional motor deficits, increased astrocyte activation, and severe CNS vascular permeability, while 129S1/SvImJ (129S1) mice remain resistant. Therefore, to investigate the potential role of genetic factors, we performed a comprehensive genetic analysis of (B6 x 129S1) F2 progeny to define quantitative trait loci (QTL) linked to the phenotypic characteristics stated above that mediate CD8 T cell-initiated BBB disruption.

**Results:**

Using single nucleotide polymorphism (SNP) markers and a 95% confidence interval, we identified one QTL (*PIFS1*) on chromosome 12 linked to deficits in motor function (SNP markers rs6292954, rs13481303, rs3655057, and rs13481324, LOD score = 3.3). In addition we identified a second QTL (*PIFS2*) on chromosome 17 linked to changes in CNS vascular permeability (SNP markers rs6196216 and rs3672065, LOD score = 3.7).

**Conclusions:**

The QTL critical intervals discovered have allowed for compilation of a list of candidate genes implicated in regulating functional deficit and CNS vascular permeability. These genes encode for factors that may be potential targets for therapeutic approaches to treat disorders characterized by CD8 T cell-mediated BBB disruption.

## Background

Disruption of the blood brain barrier (BBB) is a common feature of several devastating immune-mediated neurological disorders, including multiple sclerosis, dengue hemorrhagic fever, cerebral malaria, stroke, and acute hemorrhagic leukoencephalitis (AHLE) [1-8]. The underlying molecular mechanisms of BBB disruption observed in these conditions remain poorly understood, undermining the development of potential therapeutic approaches for human diseases characterized by BBB disruption. Our lab developed an inducible murine model of CNS vascular permeability using a variation of the Theiler’s murine encephalomyelitis virus (TMEV) model commonly used to study multiple sclerosis. In this model, peptide-specific stimulation of CNS-infiltrating CD8 T cells in vivo initiates disruption of the BBB, resulting in astrocyte activation, severe CNS vascular permeability, and functional motor deficits, followed by death within 48 hours. This peptide induced fatal syndrome (PIFS) is dependent on perforin expression, but does not rely on neutrophils, CD4 T cells, TNF-α, IFN-γ, LTβR or IL-1 [9,10].

In addition to CNS-infiltrating CD8 T cells and their effector functions, there is potential for other genetic factors to contribute to BBB disruption. C57BL/6J (B6) and 129S1/SvImJ (129S1) mice both express the H2-D^b^ class I molecule and thus are resistant to chronic TMEV infection and demyelinating syndrome [11-13]. However, despite having identical MHC class I molecules, epitope-specific CNS-infiltrating CD8 T cells, and equivalent cytotoxic T lymphocyte (CTL) perforin-mediated killing, B6 mice are highly susceptible to PIFS, whereas 129S1 mice remain resistant. This difference in susceptibility was illustrated by higher glial fibrillary acidic protein (GFAP) expression, increased CNS vascular permeability, and severe functional motor deficit in VP2_121-130_-treated B6 mice when compared to VP2_121-130_-treated 129S1 mice. Furthermore, this susceptibility is transferable with the bone marrow compartment. 129S1 mice reconstituted with B6 bone marrow exhibit severe CNS vascular permeability, microhemorrhage formation, and functional deficit when compared to 129S1 mice receiving autologous bone marrow transfer [14]. Through an initial microsatellite analysis, we have determined that the PIFS phenotype is not mediated through a single gene, but rather is likely to be a complex condition [15].

In order to provide a more comprehensive analysis and determine the location of genes that influence the PIFS phenotypes, we analyzed the F2 generation of brother-sister mated F1 animals from a B6 and 129S1 cross using QTL analysis. The quantifiable traits of astrocyte activation via GFAP expression, CNS vascular permeability via fluorescent molecule diffusion across the BBB, and functional motor deficits measured with rotarod activity were chosen for study because they differ by or exceed two standards of deviation when measured in the two parental strains, which is optimal for QTL analysis [14,16]. We identified chromosomal regions that harbored QTLs responsible for functional deficit and CNS vascular permeability. Additionally, we have identified candidate genes implicated in regulating these traits. These genes can be further investigated and may become potential targets for therapeutic approaches to ameliorate neurological disorders characterized by BBB disruption.

## Results

**Figure 1:**
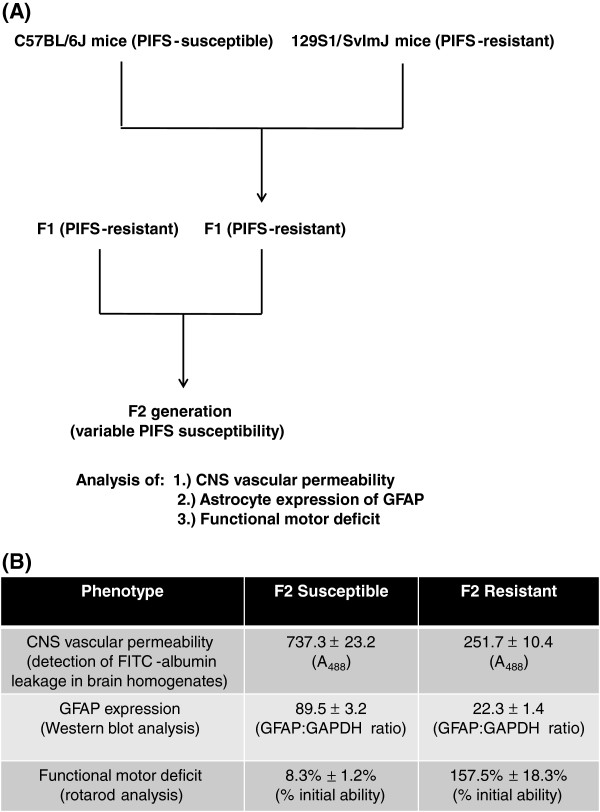
**Approach to map QTL for functional motor deficits, CNS vascular permeability, and astrocyte activation. (A)** PIFS-susceptible B6 mice were crossed with PIFS-resistant 129S1 mice to yield an F1 generation which was previously determined to be resistant to PIFS. F1 hybrid mice were then brother-sister mated to produce 303 F2 progeny with variable susceptibility to traits associated with PIFS, including functional motor deficits, astrocyte GFAP expression, and CNS vascular permeability measured by FITC-albumin leakage. DNA from 273 randomly selected F2 progeny were analyzed using Illumina Chip Technologies. SNPs with a known cM value were analyzed for the relationship between quantifiable traits (functional motor deficits, CNS vascular permeability, and astrocyte expression of GFAP) and genotype. **(B)** F2 mice displayed variable susceptibility to each of the traits associated with PIFS and were divided into susceptible and resistant groups based on whether they exhibited the trait or not. CNS vascular permeability was evaluated using a fluorescent plate reader to detect FITC-albumin leakage in brain homogenates. Samples were read at A_488_, and a threshold value of 500 was used to determine susceptibility or resistance. Astrocyte expression of GFAP was detected through Western blot analysis. Expression values were based on GFAP protein levels normalized to GAPDH. We used a threshold value of 50 to categorize F2 mice into susceptible and resistant groups. Functional motor deficit was assessed using the rotarod behavioral assay. A threshold value of 50% initial motor ability was used to categorize mice as susceptible or resistant. All values are displayed as mean ± SEM.

As shown in Figure 1A, B6 mice were crossed with 129S1 mice to yield an F1 generation, and then F1 hybrid mice were brother-sister mated to produce 303 F2 progeny. These mice were intracranially infected with TMEV and then intravenously administered VP2_121-130_ peptide 7 days later to induce PIFS. Mice were then analyzed for CNS vascular permeability, astrocyte expression of GFAP, and functional motor deficit (see Methods section). B6 mice were susceptible to each of these phenotypes, while 129S1 mice remained resistant [14]. F2 mice displayed variable susceptibility to each of these traits and were divided into susceptible and resistant groups based on whether they exhibited the trait or not. CNS vascular permeability was evaluated using a fluorescent plate reader to detect FITC-albumin leakage in brain homogenates using established methods [14,17,18]. Samples were read at A_488_, and a threshold value of 500 was used to determine susceptibility or resistance. Astrocyte expression of GFAP was detected through Western blot analysis as previously published [14,18]. Expression values were based on GFAP protein levels normalized to GAPDH. We used a threshold value of 50 to categorize F2 mice into susceptible and resistant groups. Functional motor deficit was assessed using the rotarod behavioral assay. A threshold value of 50% initial motor ability was used to categorize mice as susceptible or resistant (Additional file 1). The phenotype data for each trait is illustrated by the mean ± SEM for the F2 susceptible group and the F2 resistant group (Figure 1B). No significant sex differences were observed in the F2 mice (data not shown).

### Identification of QTL controlling quantitative traits associated with PIFS

**Figure 2:**
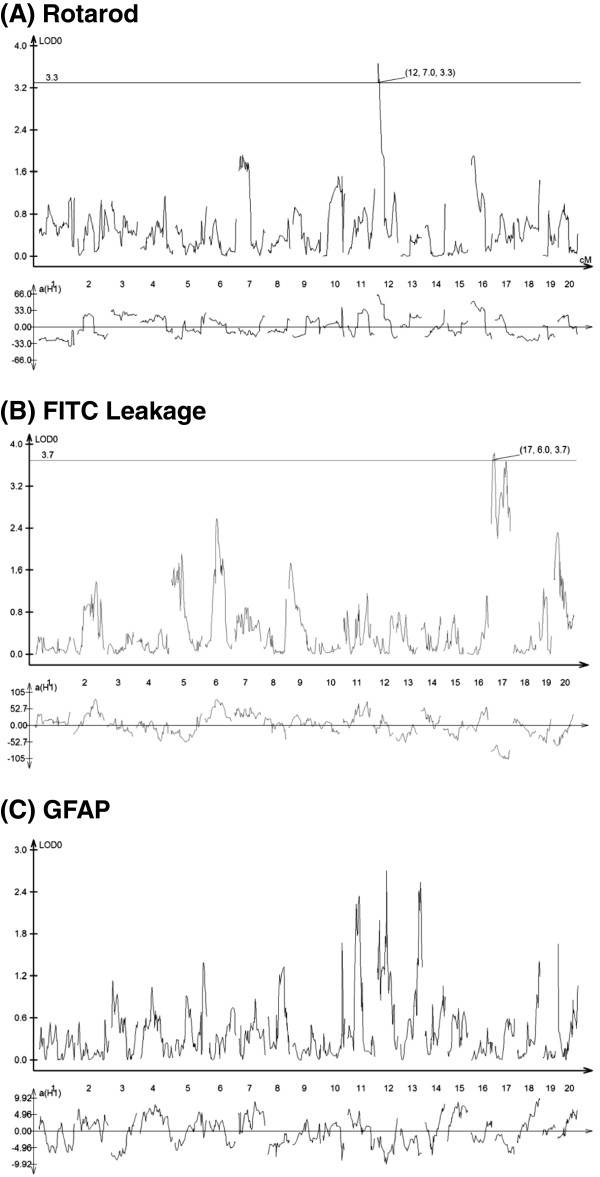
**QTL analysis of functional motor deficits, CNS vascular permeability, and astrocyte activation.** Log-likelihood plots of **(A)** functional motor deficits as measured by rotarod performance, **(B)** CNS vascular permeability as measured by leakage of FITC-albumin into the CNS, and **(C)** astrocyte activation as measured by GFAP expression. The upper graph charts the data as a LOD score. The solid black line depicts the significance threshold. The lower graph depicts the estimated QTL effects. Significant LOD scores were obtained for **(A)** functional motor deficits, which is shown to map to chromosome 12 (LOD = 3.3), and **(B)** CNS vascular permeability, which is shown to map to chromosome 17 (LOD = 3.7).

Of the 671 SNPs analyzed, the most significant linkage to susceptibility to PIFS was observed with markers on chromosomes 12 and 17, as illustrated by log-likelihood plots (Figure 2). Significant LOD scores were obtained for functional motor deficits, which mapped to chromosome 12 (LOD = 3.3) (Figure 2A), and CNS vascular permeability, which mapped to chromosome 17 (LOD = 3.7) (Figure 2B). No significant LOD score was obtained for astrocyte activation as measured by GFAP expression (Figure 2C). Parental allele distribution of the linked SNPs within the QTL intervals that mapped to the phenotypes of functional motor deficits and CNS vascular permeability were detected using 2 × 3 contingency tables comparing B6 homozygous (BB), heterozygous (BS), and 129S1 homozygous (SS) mice. The QTL location on chromosome 12 (*PIFS1*, LOD 3.3), estimated by a 95% confidence interval, was from 4.7 cM to 10.1 cM, with the peak of linkage at 6.0 cM. The QTL location on chromosome 17 (*PIFS2*, LOD 3.7), also estimated by a 95% confidence interval, was from 5.7 cM to 12.5 cM, with the peak of linkage at 10.8 cM. We found that the 129S1 strain contributed to low rotarod performance, while the B6 strain contributed to high rotarod performance on these markers (Table 1A). In addition, the 129S1 strain contributed to increased CNS vascular permeability, whereas the B6 strain contributed to reduced CNS vascular permeability in the PIFS model (Table 1B).

**Table 1:** Chi-square analysis of functional deficit and CNS vascular permeability

**A. SNP**	**Low F2s (n = 145)**			**High F2s (n = 128)**			**Chi-square test**	**Chi-square p-value**	**LOD score**
	**BB**	**BS**	**SS**	**BB**	**BS**	**SS**			
rs6292954	18	80	47	50	51	27	25.93	0.000002	3.65
rs13481303	17	83	45	46	56	26	22.71	0.000012	3.66
rs3655057	19	80	46	46	54	28	19.66	0.000054	3.30
rs13481324	21	79	45	49	52	27	20.28	0.000039	3.35
**B. SNP**	**Low F2s (n = 182)**			**High F2s (n = 91)**			**Chi-square test**	**Chi-square p-value**	**LOD Score**
	**BB**	**BS**	**SS**	**BB**	**BS**	**SS**			
rs6196216	58	88	36	20	39	32	8.24	0.016245	3.73
rs3672065	60	85	37	19	39	33	9.27	0.009706	3.79

2 × 3 contingency tables comparing B6 homozygous (BB), heterozygous (BS), and 129S1 homozygous (SS) mice with regards to (A) functional motor deficits as measured by rotarod performance and (B) CNS vascular permeability as measured by leakage of FITC-albumin into the CNS. (A) Mice displaying <50% initial ability were placed in the low F2 group, while mice displaying >50% initial ability were placed in the high F2 group. 129S1 contributes to low rotarod performance on chromosome 12 markers rs6292954, rs13481303, rs3655057, and rs13481324, while B6 contributes to high rotarod performance on these markers. (B) The low F2 group consists of mice with FITC scores <500, while the high F2 group consists of mice with FITC scores >500. B6 contributes to less CNS vascular permeability on chromosome 17 markers rs6196216 and rs3672065, while 129S1 contributes to more CNS vascular permeability on these markers.

### Potential candidate genes implicated in regulating functional deficit and CNS vascular permeability in CD8 T cell-initiated BBB disruption

Using the Mouse Genome Informatics Database resources (http://www.informatics.jax.org), we performed a search between the base pairs of the significant loci found on chromosome 12 (SNP markers rs6292954, rs13481303, rs3655057, and rs13481324) and chromosome 17 (SNP markers rs6196216 and rs3672065) in order to discover genes located in proximity with these loci. Our search revealed 34 candidate genes on chromosome 12 and 58 candidate genes on chromosome 17 (Table 2). We then prioritized these genes based on known or presumed biological function and previous studies, allowing us to narrow down this list to 6 genes of interest on chromosome 12 and 8 genes of interest on chromosome 17. We focused on genes exhibiting potential immunological, hematopoietic, apoptotic, or CNS-related functions since factors related to these processes are likely to play a role in CD8 T cell-initiated BBB disruption. We then used the Mouse Phenome Database (http://www.phenome.jax.org) to determine the number of SNPs in these genes of interest that are different between the B6 and 129S1 mouse strains as well as the location of these SNPs (Table 3).

**Table 2:** Candidate genes implicated in regulating functional deficit and CNS vascular permeability in fatal BBB disruption

**Gene**	**Chr**	**cM**	**Genome coordinates**
Nt5c1b	12	5.27	10376779-10396979 (+)
Rdh14	12	5.29	10397586-10402368 (+)
1700034J04Rik	12	5.58	11228267-11229010 (-)
4930511A02Rik	12	5.58	11445039-11451618 (+)
Gen1	12	5.58	11247732-11272593 (-)
Kcns3	12	5.58	11097008-11157648 (-)
Msgn1	12	5.58	11215188-11215754 (-)
Rad51ap2	12	5.58	11462885-11469734 (+)
Smc6	12	5.58	11272692-11326591 (+)
**Vsnl1**	**12**	**5.58**	**11332053-11443455 (-)**
Fam49a	12	5.63	12268945-12383165 (+)
Gm17337	12	6	12794791-12801091 (+)
4930519A11Rik	12	6.11	12909526-12914057 (+)
Mycn	12	6.14	12942902-12948720 (-)
**Ddx1**	**12**	**6.4**	**13226113-13255980 (-)**
Nbas	12	6.45	13275933-13590616 (+)
Fam84a	12	6.93	14154405-14158844 (-)
Gm16497	12	7.03	14268340-14292490 (+)
**Trib2**	**12**	**7.3**	**15798533-15823683 (-)**
Gm5432	12	7.28	15823809-15894961 (+)
**Lpin1**	**12**	**7.9**	**16542475-16596576 (-)**
Ntsr2	12	7.98	16660276-16667042 (+)
Greb1	12	8	16677421-16807692 (-)
2410004P03Rik	12	8.04	17011764-17018360 (-)
E2f6	12	8.04	16817771-16833549 (+)
**Kcnf1**	**12**	**8.04**	**17178906-17183694 (-)**
Pqlc3	12	8.04	16995454-17007214 (-)
**Rock2**	**12**	**8.04**	**16901784-16995080 (+)**
Atp6v1c2	12	8.06	17291527-17336165 (-)
Pdia6	12	8.06	17273351-17291576 (+)
Nol10	12	8.07	17355299-17436901 (+)
Mir3066	12	8.08	17362198-17362280 (+)
Gm19657	12	8.09	17436584-17441442 (-)
Odc1	12	8.11	17551679-17558308 (+)
**Pde10a**	**17**	**4.96**	**8718237-9179513 (+)**
1700010I14Rik	17	5.53	9181198-9201184 (+)
6530411M01Rik	17	5.97	9340584-9360701 (-)
Gm17728	17	7.33	9614925-9615323 (+)
Gm17748	17	7.33	9614805-9622259 (-)
Pabpc6	17	7.47	9859362-9862569 (-)
**Qk**	**17**	**7.75**	**10399336-10512226 (-)**
A230009B12Rik	17	7.8	10649082-10685921 (+)
**Gene**	**Chr**	**cM**	**Genome Coordinates**
Gm10513	17	7.8	11918744-11925211 (-)
Gm16168	17	7.8	10689312-10762262 (+)
Gm16169	17	7.8	11000700-11019491 (+)
**Pacrg**	**17**	**7.8**	**10595878-11033177 (-)**
**Park2**	**17**	**7.8**	**11033250-12256227 (+)**
Agpat4	17	8.33	12311570-12412511 (+)
Map3k4	17	8.42	12420487-12511526 (-)
4732491K20Rik	17	8.5	12511751-12520116 (+)
**Plg**	**17**	**8.5**	**12571475-12612250 (+)**
Slc22a3	17	8.52	12612838-12700570 (-)
C030013G03Rik	17	8.56	12659710-12672444 (+)
Slc22a2	17	8.61	12777055-12821331 (+)
Slc22a1	17	8.63	12841740-12868704 (-)
**Igf2r**	**17**	**8.64**	**12875272-12962530 (-)**
Airn	17	8.66	12934177-13052988 (+)
Gm19270	17	8.66	12926941-12946579 (+)
Gm17592	17	8.69	13030801-13031000 (-)
**Mas1**	**17**	**8.69**	**13033945-13061009 (-)**
Mrgprh	17	8.7	13068900-13070708 (+)
Pnldc1	17	8.71	13081768-13102866 (-)
Acat3	17	8.72	13116699-13133268 (-)
Mrpl18	17	8.72	13104215-13109211 (-)
Snora20	17	8.72	13115723-13115783 (+)
Tcp1	17	8.72	13108567-13117933 (+)
Acat2	17	8.73	13135756-13153613 (-)
Wtap	17	8.73	13159662-13185412 (-)
Sod2	17	8.75	13200705-13210985 (+)
Gpr31b	17	8.76	13244187-13245146 (-)
Tcp10b	17	8.76	13253970-13275347 (+)
Gm11166	17	8.78	13289526-13291338 (-)
Unc93a	17	8.78	13301483-13324657 (-)
Gm10512	17	8.81	13397908-13399077 (+)
Smok2a	17	8.82	13414054-13420524 (+)
Smok2b	17	8.82	13421718-13430055 (+)
Tcp10c	17	8.86	13547438-13570089 (+)
Rnu6	17	8.88	13624899-13625005 (-)
2700054A10Rik	17	8.9	13675419-13898832 (-)
Tcte2	17	8.9	13675419-13898832 (-)
Gm16050	17	8.91	13695065-13704059 (+)
Gm8603	17	8.91	13709618-13711010 (+)
Smok4a	17	8.91	13714322-13721299 (+)
4930488N24Rik	17	8.95	14238826-14243457 (-)
Dact2	17	8.95	14332238-14340838 (-)
Gm16046	17	8.95	13812667-13820523 (-)
Gm16049	17	8.95	13896413-13897375 (-)
Gm16052	17	8.95	13896377-13896992 (-)
Gm7168	17	8.95	14085380-14087685 (+)
Gm7356	17	8.95	14137246-14138709 (-)
Gm7358	17	8.95	14195012-14196538 (-)
**Mllt4**	**17**	**8.95**	**13897546-14043157 (+)**

Shown are a list of genes between the significant loci found on chromosome 12 (SNP markers rs6292954, rs13481303, rs3655057, and rs13481324) and chromosome 17 (SNP markers rs6196216 and rs3672065). Genes that exhibited potential **immunological, hematopoietic, apoptotic, or CNS-related functions** were categorized as genes of interest.

**Table 3:** Genes of interest potentially related to B6 and 129S1 phenotypic differences in CD8 T cell-initiated BBB disruption

**Chromosome 12 (Rotarod)**	**Proposed function**	**# SNPs different between strains**	**SNP locations**
Vsnl1	Neuronal Ca2+ sensor proteins	24	All I
Ddx1	Viral responses	2	All I
Trib2	Induces apoptosis of cells in hematopoietic origin; CD8 T cells	40	1 Cs, 1 U5, 38 I
Lpin1	Demyelination; actin cytoskeleton reorganization	531	3 Cn, 8 Cs, 27 U3, 493 I
Kcnf1	Neurotransmitter release; neuronal excitability	35	4 Cs, 7 U5, 24 U3
Rock2	Actin cytoskeleton organization; regulation of angiogenesis	330	1 Cn, 3 Cs, 5 U3, 321 I
**Chromosome 17 (CNS vascular permeability)**	**Proposed function**	**# SNPs different between strains**	**SNP locations**
Pde10a	Signal transduction; neuronal cell bodies	1268	Unspecified
Qk	Myelinization; axon ensheathment; vasculogenesis	214	12 U3, 202 I
Pacrg	Myelinization	265	2 U5, 263 I
Park2	Neuron projection; neuron death	3198	2 Cs, 1 U3, 3195 I
Plg	Apoptotic processes; vessel development	38	1 Cn, 37 I
Igf2r	Edema	7	All I
Mas1	Inflammatory responses	2	All I
Mllt4	Adherens junctions; cell junctions	7	All I

Shown are the genes of interest found on chromosome 12 and chromosome 17 along with their proposed functions. The Mouse Phenome Database (http://www.phenome.jax.org) was used to determine the number of SNPs that are different between the B6 and 129S1 mouse strains as well as their location.

(*Abbreviations:Cn* nonsynonomous codon, *Cs* synonomous codon, *U5* 5^1^ UTR variant, *U3* 3^1^ UTR variant, *I* intron-variant).

## Discussion

In this study, we investigated the role of genetic factors involved with CD8 T cell-initiated fatal BBB disruption. We previously demonstrated that despite having identical MHC class I molecules, epitope-specific CNS-infiltrating CD8 T cells, and similar levels of perforin-mediated killing, the B6 strain is highly susceptible to PIFS, while the 129S1 strain remains resistant [14,15]. This susceptibility is transferable with the bone marrow compartment, as 129S1 mice reconstituted with B6 bone marrow exhibit characteristics of the PIFS phenotype, which include severe CNS vascular permeability, microhemorrhage formation, and functional motor deficit, when compared to 129S1 mice receiving autologous bone marrow transfer [14]. Furthermore, by conducting a backcross of (129S1 × B6) F1 mice, we determined through microsatellite analysis that susceptibility to PIFS is dictated in a complex fashion [15]. Therefore, we investigated the characteristics of PIFS in F2 generation mice through analysis of three quantifiable traits, which include functional motor deficit on rotarod, activation of astrocytes, and CNS vascular permeability. Since we have shown that the means of each of these traits measured between the B6 and 129S1 mouse strains differ by greater than two standards of deviation, we expected to find a set of QTLs for each trait measured [14].

We identified one novel QTL (*PIFS1*) on chromosome 12 linked to functional motor deficits and a second novel QTL (*PIFS2*) on chromosome 17 linked to CNS vascular permeability. While causality cannot be determined in this study, we have not observed motor dysfunction without CNS vascular permeability in the PIFS model. Furthermore, we discovered that the SNP markers identified within the QTL intervals for the PIFS-resistant 129S1 strain actually contributed to low rotarod performance and more CNS vascular permeability, while the B6 strain contributed to functional motor preservation and reduced CNS vascular permeability. While this was an unexpected result, it is possible that the expression of susceptibility loci may be suppressed by the presence of other loci in the parental strain. However, since new genetic combinations can be revealed through an F2 analysis, the identification of cryptic QTL becomes possible. For example, a genetic analysis of (B6 × FVB/NJ) F2 progeny to study atherosclerosis revealed that the FVB/NJ strain, which is more resistant to atherosclerosis than the B6 strain, actually contributed to high levels of the disease [19]. Therefore, while our finding may seem surprising, it is important to note that disease-resistant strains also harbor susceptibility alleles, consistent with other genetic studies [19-21].

Identification of QTL regions that influence resistance and susceptibility to PIFS has enabled us to identify candidate genes that mediate protection from PIFS and thus are potential targets for therapy in diseases characterized by BBB disruption. Genes whose biological function had relevance to playing a role in BBB disruption were categorized as genes of interest and will be targeted first in future studies. Genes of interest pertaining to mouse functional ability on chromosome 12 include tribbles homolog 2 (*Trib2*), which has been shown to induce apoptosis of cells in hematopoietic origin. Additionally, mice homozygous for a null *Trib2* allele display increased mean percentage of CD8 T cells. Furthermore, *Trib2* has also been characterized as one of the top non-HLA markers associated with susceptibility to multiple sclerosis [22]. Considering our previous research on the critical roles of CD8 T cells and hematopoietic factors in mediating BBB disruption, *Trib2* will be a candidate for future study [9,10,14,15,17,18]. Other genes of interest include *Lpin1* and *Rock2*, which both play a role in organization of the actin cytoskeleton, *Vsnl1*, which plays a role in neuronal Ca^2+^ sensor proteins, *Kcnf1*, which plays a role in neurotransmitter release and neuronal excitability, and *Ddx1*, which plays a role in viral responses. Genes of interest on chromosome 17 that may affect CNS vascular permeability include *Pde10a*, *Qk*, *Pacrg*, *Park2*, *Plg*, *Igf2r*, *Mas1*, and *Mllt4*. These genes exhibit a variety of functions that may be involved in causing disruption of the BBB and ensuing CNS vascular permeability. For example, *Mllt4* has been shown to play a role in adherens junctions and cell junctions. *Plg* has been demonstrated to be involved in apoptotic processes and vessel development. Additionally, *Mas1* is involved in inflammatory responses, *Igf2r* is involved in edema, and *Qk* and *Pacrg* both play a role in myelinization. Furthermore, *Park2* has been shown to contribute to neuron projection as well as neuron death, and *Pde10a* has been shown to be involved in signal transduction and neuronal cell bodies (http://www.informatics.jax.org/). Furthermore, we used the Online Mendelian Inheritance in Man (OMIM) database (http://omim.org/) to investigate whether any of these genes of interest have been associated with any human neurological diseases. We found that *Vsnl1*, which is located on mouse chromosome 12, has been implicated in Alzheimer’s disease. *Qk*, located on mouse chromosome 17, has been proposed to play a role in myelin and oligodendrocyte dysfunction in schizophrenia. Additionally, *Pacrg*, also located on mouse chromosome 17, may be involved in acquired, communicating hydrocephalus (http://omim.org/).

Our comprehensive genetic analysis has allowed us to create this list of candidate genes, which will serve as a critical starting point in elucidating the underlying molecular mechanisms of BBB disruption in neurological diseases. This will be an essential first step in developing potential therapeutic approaches to treat these diseases. Validating strain differences in primary DNA sequences in candidate genes and performing bioassays to test for functional consequences of primary DNA differences will be essential in this process. It will also be essential to test candidate genes for their ability to contribute to susceptibility or resistance to functional deficit and BBB disruption in the PIFS model. Testing specific congenic strains in which potential genes of interest will be introduced will help define the importance of the QTLs detected in this study. This approach has been successful in other genetic studies [23,24]. Generation of bacterial artificial chromosome (BAC) transgenic mice using the candidate genes we have identified will also be beneficial in evaluating the importance of our novel QTLs. This will enable us to test whether strain-specific variation in one or more of these genes is responsible for causing the QTL effects we observed [25]. This method has been carried out successfully in other genetic studies of strain comparison, such as evaluating the difference in seizure susceptibility between B6 and DBA/2 mouse strains [25]. Ultimately, results obtained from these experiments may hold promise for therapeutic intervention of immune-mediated neurological diseases characterized by extensive BBB disruption.

## Conclusions

The comprehensive genetic analysis performed in this study has enabled us to identify QTL linked to functional motor deficits and CNS vascular permeability, which are both devastating characteristics associated with CD8 T cell-initiated BBB disruption. Furthermore, identification of these critical QTL intervals has revealed a list of candidate genes implicated in regulating these traits. These findings are important because they will set the stage for future research to identify novel factors encoded by these genes that affect immune-mediated BBB disruption. This may ultimately hold promise for devising potential therapeutic approaches to treat the numerous immune-mediated neurological disorders characterized by BBB disruption.

## Methods

### Animals

Male and female C57BL/6J (B6) (stock# 000664) and 129S1/SvImJ (129S1) (stock# 002448) mice were obtained from Jackson laboratories (Bar Harbor, ME). (B6 × 129S1) F1 hybrids were brother-sister mated to produce (B6 × 129S1) × (B6 × 129S1) F2 progeny. All F1 and F2 mice were produced in the animal colony at the University of Cincinnati (Cincinnati, OH). In order to obtain large cohorts, mice were tested at 4–7 months of age. All experiments were approved by the Institutional Animal Care and Use Committee of the University of Cincinnati and Mayo Clinic.

### Induction of CNS vascular permeability using the PIFS model

CNS vascular permeability was induced as previously described [15,17,18]. Briefly, all mice were intracranially infected with 2 × 10^6^ PFU Daniel’s strain of TMEV. 7 days post-TMEV infection, during the peak of CD8 T cell expansion, mice were intravenously administered 0.1 mg VP2_121-130_ (FHAGSLLVFM) peptide [15].

### Rotarod

In order to evaluate functional motor deficits, mice were placed on the Rotamex-5 rotarod apparatus (Columbus Instruments) increasing from 5–40 RPMs over 7 minutes. Mice were trained twice daily for 3 consecutive days. The motor performance of mice was then assessed the day following this training period in order to establish baseline ability, which was considered the average of two trials. Mice were then injected with VP2_121-130_ peptide to induce CNS vascular permeability and their motor performance was assessed again 24 hours later. Final scores were depicted as percent initial motor ability (day 2 average/day 1 average × 100).

### Fluorescein isothiocyanate (FITC)-albumin permeability assay

Mice were given an intravenous injection of 10 mg FITC-albumin (Sigma #A9771) 23 hours post-VP2_121-130_ peptide administration to induce PIFS. Whole brains were harvested one hour after administration of FITC-albumin and frozen on dry ice. Brain tissue samples were homogenized in radioimmunoprecipitation assay (RIPA) buffer [10 mmol/L Tris, 140 mmol/L NaCl, 1% Triton X-100, 1% Na deoxycholate, 0.1% SDS and protease inhibitor cocktail (Pierce #78410) pH 7.5] and spun for 15 min at 10,000 RPM. Protein concentration was assessed using the BCA protein assay (Pierce #23223). Samples were then normalized for protein content and read on a fluorescent plate reader at 488 nm excitation and 525 nm emission to detect FITC-albumin leakage into the brain. Data was collected using SpectraMax software (Molecular Devices).

### Western blot for glial fibrillary acidic protein (GFAP) detection

Normalized protein samples from the FITC-albumin permeability assay were also used for western blot analysis. In order to detect GFAP, 10 ug of protein was loaded per well on 4–20% precise protein gels (Pierce #25244) with BupH Tris-HEPES-SDS running buffer (Pierce #28398). Tris transfer buffer [400 mmol/mL Tris base, 70 mmol/mL glycine, 10% methanol] was used to transfer gels onto Immun-Blot PVDF membranes (Biorad #162-0177). GFAP was detected by staining with mouse anti-GFAP (1:1000 BD Pharmingen #556329) and goat anti-mouse IgG conjugated to horseradish peroxidase (Sigma #A3682). Scion Image Software (Scion Corp) was used to analyze Western blot films. Values were based on GFAP protein levels normalized to GAPDH [14,18].

### Quantitative trait linkage analysis

DNA from 273 F2 progeny (124 males; 149 females) were analyzed on 2 SNP chips using Illumina Chip Technologies. Two B6 and 2 129S1 mice were included as controls. In order to map genes controlling susceptibility to PIFS in the B6 × 129S1 F2 model, we constructed a genetic linkage map of the mouse genome by analyzing 273 randomly selected mice using Illumina Chip Technologies. Of the 1449 analyzable SNPs, 874 were found to be unique between the two strains. 671 of these SNPs had a known cM value and thus were analyzed for the relationship between quantifiable traits and genotype [26]. Based on previous studies, we used functional motor deficits measured by rotarod, CNS vascular permeability measured by FITC-albumin leakage, and astrocyte activation measured by GFAP expression as quantifiable traits predictive of susceptibility to PIFS [14,15]. Threshold values were generated using the WinQTL Cartographer with 1,000 permutations of our data set to correct for multiple testing [27]. Interval mapping was used to identify significant linkage using a critical value of p = 0.05 and a 95% confidence interval (Additional file 2). A chi-square test statistic for each SNP marker showing linkage within a QTL interval was obtained using 2 × 3 contingency tables to demonstrate the distribution of parental strain alleles within the F2 population using the quantitative traits of functional motor deficits, CNS vascular permeability, and astrocyte activation. Since F2 mice displayed variable susceptibility, they were divided into two groups, low F2s and high F2s, based on whether or not they exhibited functional motor deficits or CNS vascular permeability. For functional motor deficits, low F2s were defined as those mice displaying <50% initial ability, while high F2s were defined as mice displaying >50% initial ability. For CNS vascular permeability, mice with FITC scores <500 were placed in the low F2 group while mice with FITC scores >500 were placed in the high F2 group for analysis. Since data were scored in a binomial fashion, we used a chi-square test, which is a nonparametric approach, to determine significance. We identified the critical interval for significant QTL and performed a search using http://www.informatics.jax.org to compile a list of candidate genes. These genes were then prioritized based on their known or presumed biological function. We then used the Mouse Phenome Database (http://www.phenome.jax.org) to determine the number of SNPs in these genes of interest that are different between the B6 and 129S1 mouse strains as well as the location of these SNPs. These genes will set the stage for future experiments on gene expression and strain differences that may be related to the mechanism of CD8 T cell-initiated BBB disruption.

### Availability of supporting data

The data sets supporting the results of this article are included as additional files.

## Abbreviations

BBB: Blood–brain barrier; TMEV: Theiler’s murine encephalomyelitis virus; PIFS: Peptide-induced fatal syndrome; QTL: Quantitative trait loci; SNP: Single nucleotide polymorphism; FITC: Fluorescein isothiocyanate; GFAP: Glial fibrillary acidic protein.

## Competing interests

The authors declare that they have no competing interests.

## Authors’ contributions

HLJ performed rotarod behavioral assays, assisted with acquiring DNA and analyzing data, constructed the figures, and prepared the manuscript. LMH prepared DNA to be analyzed on the SNP chips, analyzed data using WinQTL Cartographer, and assisted with construction of figures. YC performed rotarod behavioral assays, assisted with acquiring DNA, and carried out western blots and FITC-albumin permeability assays. AJB assisted with analysis and interpretation of data. RJB assisted with analysis and interpretation of data and manuscript preparation. TNF contributed to revising the manuscript. IP participated in experimental designs and revising the manuscript. AJJ performed intracranial and intravenous injections, participated in acquiring DNA, and was involved in experimental design and preparing the manuscript. All authors read and approved the final manuscript.

## Supplementary Material

Additional file 1 **Shown are the raw data obtained for CNS vascular permeability, as measured by assaying FITC-albumin leakage in brain homogenates, astrocyte expression of GFAP, as detected by Western blot analysis, and functional motor deficit, as assessed using the rotarod behavioral assay.** Mice were divided into susceptible and resistant groups using the criteria stated in the text. Raw data values were used to determine mean ± SEM for each trait. Click here for file

Additional file 2 **Shown are the raw data used to analyze the SNPs with known cM values for the relationship between quantifiable traits and genotype using WinQTL Cartographer.** Mouse identification numbers are shown across the top. SNP markers, chromosome numbers, cM values, and genotype data are included. For genotype, 0 = homozygous for B6, 1 = heterozygous, and 2 = homozygous for 129S1. Raw data for each quantifiable trait are included in a separate tab. Click here for file
